# Assessment of heart rate variability (HRV) in subjects with type 2 diabetes mellitus with and without diabetic foot: correlations with endothelial dysfunction indices and markers of adipo-inflammatory dysfunction

**DOI:** 10.1186/s12933-021-01337-z

**Published:** 2021-07-14

**Authors:** Antonino Tuttolomondo, Alessandro Del Cuore, Alessandro La Malfa, Alessandra Casuccio, Mario Daidone, Carlo Domenico Maida, Domenico Di Raimondo, Tiziana Di Chiara, Maria Grazia Puleo, Rosario Norrito, Giovanni Guercio, Antonio Pinto

**Affiliations:** 1 Internal Medicine and Stroke Care Ward, University of Palermo, Palermo, Italy; 2 Department of Health Promotion, Mother and Child Care, Internal Medicine and Medical Specialties (ProMISE), University of Palermo, Palermo, Italy; 3 Department of Surgical Oncological and Stomatological Disciplines, University of Palermo, Palermo, Italy

## Abstract

**Background:**

Some studies have suggested that patients with diabetes and foot complications have worse cardiovascular and cerebrovascular risk profiles, higher degrees of endothelial dysfunction and arterial stiffness and a higher inflammatory background than patients with diabetes without diabetic foot complications. Patients with diabetes mellitus have an alteration in the sympathovagal balance as assessed by means of heart rate variability (HRV) analysis, which is also related to the presence of endothelial dysfunction. Other studies suggest a possible role of inflammation coexisting with the alteration in the sympathovagal balance in favor of the atherosclerotic process in a mixed population of healthy subjects of middle and advanced age.

**Aims:**

The aim of this study was to evaluate the degree of alteration of sympathovagal balance, assessed by HRV analysis, in a cohort of patients with diabetes mellitus with diabetic foot and in control subjects without diabetic foot compared with a population of healthy subjects and the possible correlation of HRV parameters with inflammatory markers and endothelial dysfunction indices.

**Methods:**

We enrolled all patients with diabetic ulcerative lesions of the lower limb in the Internal Medicine with Stroke Care ward and of the diabetic foot outpatient clinic of P. Giaccone University Hospital of Palermo between September *2019 and July 2020. 4-h ECG Holter was performed. The following time domain HRV measures were analyzed: average heart rate, square root of the mean of successive differences of NN (RMSSD), standard deviation or square root of the variance (SD), and standard deviation of the means of the NN intervals calculated over* a five-minute period (SDANN/5 min). The LF/HF ratio was calculated, reactive hyperemia was evaluated by endo-PAT, and serum levels of vaspine and omentin-1 were assessed by blood sample collection.

**Results:**

63 patients with diabetic foot, 30 patients with diabetes and without ulcerative complications and 30 patients without diabetes were enrolled. Patients with diabetic ulcers showed lower mean diastolic blood pressure values than healthy controls, lower MMSE scores corrected for age, lower serum levels of omentin-1, lower RHI values, higher body weight values and comparable body height values, HF% and LF/HF ratio values. We also reported a negative correlation between the RHI value and HRV indices and the expression of increased parasympathetic activity (RMSDD and HF%) in subjects with diabetic foot and a statistically significant positive correlation with the LF/HF ratio and the expression of the sympathovagal balance.

**Discussion:**

Patients with diabetic foot show a higher degree of activation of the parasympathetic system, expressed by the increase in HF values, and a lower LF/HF ratio. Our findings may corroborate the issue that a parasympathetic dysfunction may have a possible additive role in the pathogenesis of other vascular complications in subjects with diabetic foot.

## Background

Among diabetic vascular complications, foot ulcers are the major cause of hospitalization in patients with diabetes and a significant cause of health care costs (over 20%–40% of health care resources have been reported to be related to foot care related to diabetes).

Several studies also indicate that foot ulcers in patients with diabetes are related to high mortality. In fact, diabetic foot is a major cause of morbidity in patients with diabetes, and the mortality rate is approximately double that of patients without a foot ulcer [1–4]. Some studies also suggest that patients with diabetes and foot complications, compared to patients with diabetes without diabetic foot complications, have a worse cardiovascular and cerebrovascular risk profile, a higher degree of endothelial dysfunction and arterial stiffness and a greater inflammatory background [2, 3, 5].

Some authors also reported that patients with diabetes mellitus have an alteration in the sympathovagal balance assessed by means of heart rate variability (HRV) analysis, which is also related to the presence of endothelial dysfunction [6]. Finally, other studies suggest a possible role of inflammation coexisting with the alteration in the sympathovagal balance favoring the atherosclerotic process in a mixed population of healthy subjects of middle and advanced age [7, 8].

Nevertheless, a few studies [6, 7] have analyzed the degree of alteration of the autonomic nervous system in patients with diabetes and foot complications compared to patients with diabetes without diabetic foot complications. To the best of our knowledge, no study has evaluated the possible association between the inflammatory background of patients with diabetic foot and alterations in the autonomic nervous system.

On this basis, the aim of our study was to evaluate whether patients with diabetes and foot ulcers present a higher degree of sympathovagal imbalance (in favor of activation of the sympathetic nervous system) than diabetic subjects without foot complications to highlight whether this alteration may be associated with a more extensive inflammatory pathway and a higher degree of endothelial dysfunction in these subjects than in those without complications.

Thus, the aim of this study project was to evaluate the degree of alteration of the sympathovagal balance, assessed by HRV analysis, in a cohort of patients with diabetes mellitus with diabetic foot and in control subjects without diabetic foot compared with a population of healthy subjects and the possible correlation of HRV parameters with inflammatory markers and endothelial dysfunction indices.

### Aims of the study

1. The first aim of our study was to evaluate the degree of autonomic nervous system dysfunction by analyzing heart rate variability (HRV) in a cohort of patients with diabetes with and without foot complications compared to a sample of healthy control subjects.
2. The second aim was to evaluate any correlation between HRV parameters (SDNN and the LF/HF ratio) and adipoinflammatory dysfunction markers and endothelial dysfunction indices in the same cohort of patients.

## Materials and methods

We enrolled all patients with diabetic ulcerative lesions of the lower limb of the diabetic foot outpatient clinic of the Surgery Department of P. Giaccone University Hospital of Palermo and those admitted to the Internal Medicine with Stroke Care ward of P. Giaccone University Hospital of Palermo between September 2019 and July 2020.

During this period, patients with diabetes with no mention of ulcerative complications were also recruited as controls afferent at our ward for clinical complications related to diabetes (glycometabolic decompensation, hypoglycemia, skin lesions) and a population of healthy controls without diabetes admitted to our ward for other causes.

All patients provided informed consent to take part in the study and for data disclosure in accordance with the principles of the 2001 Helsinki Declaration.

All patients with inflammatory or infectious diseases, autoimmune and/or rheumatic diseases, neoplasms, hematological diseases, severe renal or hepatic insufficiency, fever, treatment with anti-inflammatory drugs, and recent hospitalization in the last month were excluded from the study.

Diabetic foot also called Diabetic foot syndrome (DFS) is defined, according to the WHO, as an ulcerative lesion of the foot (including the ankle) related to neuropathy and to different degrees of ischemia and infection [8]. Ulcerative foot injury is defined as a continuous lesion of the skin that takes more than 14 days to heal [9].

Each subject with diabetic foot will therefore be matched by age (± 3 years) and sex with a subject without diabetic foot and a healthy subject.

Diabetic distal symmetric polyneuropathy (DSPN) was assessed by collecting the patient’s medical history, by clinical evaluation and by diagnostic tests such as pinprick and temperature sensation for evaluation of small-fiber function and 10-g monofilament, and ankle reflex for Large-fiber function [10, 11]. DSPN was defined by the presence of symptoms and/or signs of peripheral nerve dysfunction in people with diabetes after the exclusion of other cause [11].

To evaluate the symptoms of neuropathy, the Neuropathy Symptom Score (NSS) [10] was used, which is generally used in clinical practice and has shown high validity and sensitivity [11].

A careful objective examination of the lower limb was carried out to look for the presence of the following characteristics: hammer or claw foot, Charcot deformity, limit of the big toe, prominent metatarsal heads, hallux valgus, bony prominences and abnormal ankle angle. The big toe was measured with a goniometer.

The diagnosis of type 2 diabetes mellitus was based on the revised American Diabetes Association (ADA) criteria, using random blood glucose values > 126 mg/dL or using a clinical algorithm that considered the age of onset, symptoms, present weight, family history, initiation of insulin treatment and history of ketoacidosis [12].

Hypertension was defined using the ESC 2018 criteria [13].

Dyslipidemia was defined by triglyceride levels ≥ 150 mg/dl and HDL cholesterol levels < 40 mg/dl based on the patient’s sex [14].

### Laboratory analysis

Clinical and anthropometric data were collected at the time of enrollment. Patients were classified as obese (BMI ≥ 30 kg/m^2^), overweight (BMI 25–29.9 kg/m^2^), or normal weight (BMI 18.5–24.9 kg/m^2^).

At the time of enrollment, a blood sample was taken to assess the levels of ALT, triglycerides, blood sugar, total cholesterol and HDL cholesterol.

### Evaluation of cognitive performance

The Mini-Mental State Examination (MMSE) was administered, and a questionnaire with 11 questions was used to evaluate the 6 major cognitive areas (language, attention, orientation, memorization, repetition and calculation). The maximum score is 30, while MMSE values below 24 (23 or lower) appear to be suggestive of “mild cognitive impairment” [15].

### Evaluation of endothelial indices

The principle of RH-PAT has been previously described by some studies. Briefly, a blood pressure cuff was placed on an upper limb, while the contralateral arm was used as a control. The PAT probe was placed on one finger of each of the two hands. After a period of 5 min of control measurement, the blood pressure cuff was inflated to 60 mmHg above the previously measured systolic pressure or up to 200 mmHg for 5 min, after which it was deflated to induce reactive hyperemia. The RH-PAT data were analyzed digitally using the Endo-PAT2000 software version 3.0.4. The RH-PAT index reflects the extent of reactive hyperemia and was calculated as the ratio of the mean PAT signal amplitudes above the first minute of initial measurement and 1.5 min of measurement after cuff deflation (A: control arm; C: occluded arm) divided by the average of the PAT signal amplitudes over a 2.5-min period prior to inflation of the blood pressure cuff (B: control arm; D: occluded arm). This RH-PAT index, called the reactive hyperactivity index (RHI), is expressed by the formula RHI = (C/D)/(A/B) × basal correction.

### Evaluation of the sympathovagal balance

For the execution of the 24-h ECG Holter, Sorin Spiderview Digital Holter Recorder devices were used, and the analysis of the collected data was carried out through the ELA Medical SyneScope version 3.10 program. The device was placed between 8:30 and 9:30 in the morning and worn by the patient for 24 h. The Holter recorder involves the placement of ten electrodes in the precordial region. During monitoring, the patient was asked to carry out normal daily activity while avoiding intense physical exercise. The trace was recorded on a special card contained in the device and subsequently analyzed on the computer through 3 recording channels. The analysis, carried out by a single operator trained in the use of the software, initially provided for the distinction between artifacts, normal beats and ventricular beats and, subsequently, the assessment by the operator of the events selected by the computer.

By event, we mean:

- Minimum and maximum RR
- Minimum and maximum HR
- Supraventricular tachycardia (SVT) (HR > 150 bpm)
- Ventricular tachycardia (TV) (HR > 50 bpm)
- Bradycardia (HR < 45 bpm)
- Pause (NN greater than 2500 ms)
- Missing beats
- Supraventricular (SVBE) and ventricular (EVB) ectopic beats, single, in
- Couple and in short run (three to fifteen beats)
- SVBE and EVB at two or three times
- Instability NN
- Acceleration and/or deceleration NN

The operator reserved the right to reevaluate the remaining part of the track, even if one or more events were not automatically detected, for the correct classification.

ECG Holter monitoring was considered invalid in the following cases:

- Recording duration < 22 h
- Number of artifacts and ectopic beats > 20% of the total number of beats (in the presence of ectopic beats, both the NN interval represented by the copula and that represented by the post-extrasystolic pause were excluded from the final analysis)
- For each monitoring, the HRV measurements of the time domain and frequency in the day (8–21 h), night (23–6 h) and 24 h were taken into consideration.
- The following time domain HRV measures were analyzed:
- Average heart rate
- Square root of the mean of successive differences of NN (RMSSD)
- Standard deviation or square root of the variance (SD)
- Standard deviation of the means of the NN intervals calculated over a five-minute period (SDANN/5 min).
- The following frequency domain HRV measures were analyzed:
- High frequency (0.15–0.4 Hz) (HF%)
- Low frequency (0.04–0.15 Hz) (LF%)
- LF/HF ratio: ratio of Low Frequency to High Frequency.

### Biochemical analysis

As expression of adipo-inflammatory dysfunction, serum levels of vaspine and omentin-1 were assessed by blood sample collection. The serum was obtained by centrifugation at 3000 rpm for 10 min and stored at a temperature of −80 °C. All samples were analyzed in the same analytical session. Vaspine serum concentrations were measured using an ELISA kit (BioVendor, BioVendor Laboratory Medicine, Inc. Czech Republic) (detection range: 0.1–1000 ng/ml; sensitivity: < 1 ng/ml). The serum levels of omentin-1 were evaluated using an ELISA kit (Human Omentin EIA, Raybiotech) (detection range: 0.031–2 ng/ml; sensitivity: 0.01 ng/ml Atlanta, USA).

### Statistical analysis

Quantitative and qualitative statistical analyses of the data, including descriptive statistics, were performed for all items. Continuous variables are expressed as the mean values ± standard deviations (SDs), unless otherwise specified. The basic significant differences between the groups were evaluated using the chi-square test or Fisher’s test, as needed, for the categorical variables. The univariate analysis of variance (ANOVA) was performed for parametric variables, and post hoc analysis with the Tukey test was used to determine whether there were pairwise intragroup differences. Multivariable logistic regression analysis examined the correlation between patient characteristics (independent variables as demographic and clinical variables resulted significant at univariate analysis), and patient groups (dependent variable, diabetic subjects and subjects with diabetic foot versus healthy subjects). In addition, odds ratios (ORs) and their 95% confidence intervals (CIs) were calculated and adjusted for drug therapy as a covariate. Pearson’s correlation analysis was conducted to evaluate the association between endothelial dysfunction indices and the clinical-laboratory-instrumental alteration variant in the various groups. Data were analyzed using SPSS Software version 22.0 (IBM Corp., Armonk, NY, USA). P-values below 0.05 were considered statistically significant.

## Results

Between September 2019 and August 2020, 63 patients with diabetic foot were enrolled at the Internal Medicine with Stroke Care ward of the University Hospital of Palermo (Italy).

We also enrolled a control group of 30 patients with diabetes and without ulcerative complications and 30 patients without diabetes admitted to our internal medicine ward for causes other than diabetes or its related complications.

The laboratory, demographic and general variables of patients with diabetes with and without ulcerative foot ulcer lesions and healthy control subjects are presented in Table 1.

**Table 1:** Demographic and clinical variables in healthcare subjects and patients with or without diabetic foot

Variables	DFS (n = 63)	NDFS (n = 30)	Healthy (n = 30)	p^a^
Sex M/F	45/18	11/19	14/16	0.003
Hypertension (n/%)	58/92	24/80	14/46	< 0.0005
Cardiovascular events (n/%)	24/38	12/40	20/66	0.028
Stroke (n/%)	13/20	4/13	3/10	0.380
Dyslipidemia (n/%)	45/71	18/60	24/80	0.231
Smoker (n/%)	23/36	10/33	25/83	< 0.0005
Beta blocker (n/%)	20/31	13/43	12/40	0.503
Calcium channel blocker (n/%)	27/42	13/43	12/40	0.958
ACE inhibitor or ARBs (n/%)	26/41	13/43	12/40	0.965
Statin (n/%)	47/74	20/66	25/83	0.331
Antiplatelet (n/%)	28/44	10/33	13/43	0.580
GLP-1 agonist or DPP4 inhibitors (n/%)	1/1	5/16	0/0	0.002
Sulfonylurea (n/%)	1/1	11/36	0/0	< 0.0005
Metformin (n/%)	10/15	16/53	0/0	< 0.0005
Insulin (n/%)	63/100	6/20	0/0	< 0.0005

DFS, diabetes foot syndrome; ARBs, Angiotensin II receptor blockers

GLP-1: *Glucagon-like peptide*-*1* receptor; DPP4 inhibitors, Inhibitors of dipeptidyl peptidase 4

^a^p, intergroup differences among patient groups (chi-square test or Fisher’s test, as needed)

Diabetic subjects with ulcerative lesions of the lower limb showed higher body height values than the diabetic controls (168.33 ± 7.19 cm vs 161.97 ± 20.12 cm; p = 0.042) and HF (21.23 ± 14.68 vs 11.10 ± 11.58; p = 0.002), lower values of the LF/HF ratio (1.63 ± 1.66 vs 3.18 ± 2.82; p = 0.001), and lower RHI values (1.60 ± 0.33 vs 2.01 ± 0.69; p < 0.0005), lower serum levels of omentin (18.95 ± 3.30 ng/ml vs 21.80 ± 5.79 ng/mL; p = 0.014) and comparable mean diastolic blood pressure values (71.14 ± 11.30 mmHg vs 75.33 ± 10.08 mmHg; p = 0.262) and body weight (84.25 ± 14.10 kg vs 87.10 ± 27.82; p = 1.00) (see Table 2).

**Table 2:** Univariate analysis results in healthcare subjects compared to patients with and without diabetic foot

Variables	DFS (n = 63)	NDFS (n = 30)	Healthy (n = 30)	p^a^
Age (years) (mean ± SD)	66.57 ± 9.54	61.20 ± 9.40	64.83 ± 6.48	0.08
SBP (mmHg) (mean ± SD)	132.81 ± 13.81	130.83 ± 17.62	128.00 ± 10.38	0.39
DBP (mmHg) (mean ± SD)	71.14 ± 11.30^b^	75.33 ± 10.08	77.66 ± 11.04	0.02
Height (cm) (mean ± SD)	168.33 ± 7.19^c^	161.96 ± 20.12	163.93 ± 5.74	0.03
Weight (kg) (mean ± SD)	84.25 ± 14.10^b^	87.10 ± 27.82^b^	73.06 ± 9.92	0.005
BMI (kg/m^2^) (mean ± SD)	29.54 ± 4.23	30.78 ± 6.67	28.08 ± 4.19	0.11
RHI (mean ± SD)	1.60 ± 0.33^bc^	2.01 ± 0.69	2.20 ± 0.38	< 0.0005
MMSE (mean ± SD)	26.77 ± 3.44	27.03 ± 2.53	27.96 ± 1.24	0.16
MMSE corrected for age (mean ± SD)	26.57 ± 3.33^b^	27.16 ± 2.38	28.14 ± 1.15	0.03
HbA_1c_ (%) (mean ± SD)	8.07 ± 1.26	7.74 ± 1.71	–	0.29
RMSDD (ms) (mean ± SD)	81.46 ± 95.22	46.23 ± 53.93	86.11 ± 74.04	0.10
SDNN/5 min (ms) (mean ± SD)	66.39 ± 23.97	59.40 ± 16.19	71.75 ± 22.52	0.09
SD (ms) (mean ± SD)	87.83 ± 50.65	85.69 ± 37.72	101.38 ± 47.07	0.34
LF% (mean ± SD)	19.63 ± 11.08	24.06 ± 13.64	55.35 ± 222.94	0.33
HF% (mean ± SD)	21.23 ± 14.68^c^	11.10 ± 11.58	14.70 ± 11.15	0.002
LF/HF (mean ± SD)	1.63 ± 1.66^c^	3.18 ± 2.82^b^	1.07 ± 0.44	< 0.0005
Omentin-1 (ng/mL) (mean ± SD)	18.95 ± 3.60^bc^	21.80 ± 5.79^b^	31.16 ± 4.53	< 0.0005
Vaspine (mean ± SD)	0.20 ± 0.05	0.21 ± 0.58	0.19 ± 0.03	0.23

SBP, systolic blood pressure; DBP, diastolic blood pressure; BMI, body mass index; RHI, reactive hyperemia index; MMSE, Mini Mental State Examination; RMSSD, root mean square of the successive differences in 5 min; SDNN, standard deviation of the NN (R-R) intervals; SD, standard deviation; LF, low frequency; HF, high frequency; LF/HF, low frequency/high frequency

^a^p, intergroup differences among patient groups, univariate analysis of variance (ANOVA) and *post hoc* analysis with the Tukey test to determine pairwise intragroup differences

^b^vs healthy subjects, ^c^vs NDFS

Patients with diabetic ulcers compared to healthy controls showed lower mean diastolic blood pressure (71.14 ± 11.30 mmHg vs 77.66 ± 11.04 mmHg; p = 0.025), lower MMSE scores corrected for age (26.77 ± 3.44 vs 28.14 ± 1.15; p = 0.038), lower serum levels of omentin (18.95 ± 3.30 ng/ml vs 31.16 ± 4.53 ng/ml; p = 0.001), lower RHI values (1.60 ± 0.33 vs 2.20 ± 0.38; p = 0.00), higher body weight values (84.25 ± 14.10 kg vs 73.06 ± 9.92; p = 0.05) and comparable body height values (168.33 ± 7.19 cm vs 163.933 ± 5.74 cm; p = 0.263), HF% (21.23 ± 14.68 vs 14.70 ± 11.15; p = 0.82) and LF/HF ratios (1.63 ± 1.66 vs 1.07 ± 0.44; p = 0.59) (see Table 2)..

Diabetic control patients showed lower serum levels of omentin (21.80 ± 5.79 ng/ml vs 31.16 ± 4.53 ng/ml; p < 0.0005), higher LF/HF ratio values (3.18 ± 2.82 vs 1.07 ± 0.44; p < 0.0005), comparable RHI values (2.01 ± 0.69 vs 2.20 ± 0.38; p = 0.315), comparable age-corrected MMSE scores (27.16 ± 2.38 vs 28.14 ± 1.15; p = 0.499) and comparable mean diastolic blood pressure values (75.33 ± 10.08 mmHg vs 77.66 ± 11.04 mmHg; p = 1.00) compared to the healthy controls (see Table 2).

Multiple regression analysis with the regard of diabetic foot presence (DFS) compared to healthy control subjects showed a significant negative association between diabetic foot and serum levels of omentin (B = − 0.56; p < 0.0005) and a positive association between diabetic foot and hypertension (B = 3.96; p = 0.01) (see Table 3).

**Table 3:** Multiple regression analysis in patients with and without DFS compared to healthcare subjects

Variables	DFS				Non DFS			
	B	p	Exp (B)	95% CI per Exp(B)	B	p	Exp(B)	95% CI per Exp(B)
RHI	− 2.10	0.15	0.12	0.007–2.15	0.55	0.64	1.74	0.16–18.07
Hypertension	3.96	0.01	52.64	2.14–1293.08	2.25	0.13	9.53	0.51–177.74
Cardiovascular events	− 2.32	0.12	0.09	0.005–1.83	− 1.89	0.19	0.150	0.009–2.62
Smoker	− 1.52	0.27	0.21	0.01–3.37	− 0.65	0.62	0.52	0.03–7.28
MMSE corrected for age	− 0.23	0.48	0.79	0.40–1.53	− 0.18	0.58	0.82	0.427–1.60
HF%	0.53	0.43	1.05	0.92–1.20	− 0.002	0.93	0.99	0.87–1.13
LF/HF (%)	1.25	0.86	3.48	0.43–0.74	1.50	0.03	4.50	1.14–18.24
Omentin-1 (ng/mL)	− 0.56	< 0.0005	0.56	0.437–0.742	− 0.31	0.003	0.72	0.58–0.89

Reference: Healthy subjects

DFS, diabetic foot syndrome; RHI, reactive hyperemia index; MMSE, Mini Mental State Examination; RMSSD HF, high frequency; LF/HF, low frequency/high frequency

Multiple regression analysis with regard of diabetes without foot ulcerations (Non DFS) compared to healthy control subjects showed a significant negative association between Non DFS and serum levels of omentin (B =− 0.31 p < 0.003) and a positive association between Non DFS and LH/HF (B = 1.50; p = 0.03) (see Table 3).

Multiple regression analysis with regard of diabetic foot presence (DFS) vs diabetes without foot ulcerations showed a significant positive association between male sex and DFS (B = 4.17, p < 0.0005) and a negative association between DFS and omentin (B = − 0.24; p = 0.009) and peripheral artery disease (B. − 0.13; p: = 0.002). AND RHI (B = − 2.65; p = 0.007) (see Table 4).

**Table 4:** Multiple regression analysis in patients without DFS compared to patients with DFS

Variables	DFS			
	B	p	Exp(B)	95% CI for Exp(B)
Sex	4.17	< 0.0005	64.9	7.7−546.8
Peripheral arterial disease (mmHg)	− 0.13	0.002	0.87	0.80−0.95
Weight (kg)	− 0.03	0.10	0.96	0.92−1.08
RHI	− 2.65	0.007	0.07	0.01−0.48
Hypertension	1.70	0.15	5.52	0.51−59.2
Cardiovascular events	− 0.42	0.57	0.65	0.15−2.84
Smoker	− 0.87	0.27	0.42	0.09−2.02
MMSE corrected for age	− 0.04	0.76	0.95	0.69−1.30
HF (Hz)	0.05	0.17	1.06	0.98−1.14
LF/HF (%)	− 0.25	0.23	0.77	0.51−1.18
Omentin-1 (ng/mL)	− 0.24	0.009	0.78	0.65−0.94

Reference: NDFS

DFS, diabetic foot syndrome; RHI, reactive hyperemia index; MMSE, Mini Mental State Examination; RMSSD, root mean square of the successive differences; HF, high frequency

LF/HF, low frequency/high frequency; NDFS, non diabetic foot syndrome: diabetic subjects without foot ulcerations

Pearson’s correlation analysis in patients with diabetic foot (DFS) showed a statistically significant negative correlation between the RHI and RMSDD (Pearson index − 0.47; p = 0.0001), SD (Pearson index − 0.374; p = 0.002), and HF% (Pearson index − 0.395; p = 0.001) and a statistically significant positive correlation between the RHI and the LF/HF ratio (Pearson index 0.280; p = 0.026) (see Table 5).

**Table 5:** Pearson correlation between the RHI and other variables in patients with DFS

		RHI
RHI	Pearson Correlation	1
	Sig. (2-tailed)	
	N	63
MMSE corrected for age	Pearson Correlation	− 0.047
	Sig. (2-tailed)	0.717
	N	63
RMSSD (ms)	Pearson Correlation	0.470
	Sig. (2-tailed)	0.0001
	N	63
SDANN5 MIN (ms)	Pearson Correlation	− 0.158
	Sig. (2-tailed)	0.216
	N	63
SD (ms)	Pearson Correlation	0.374
	Sig. (2-tailed)	0.002
	N	63
LF (%)	Pearson Correlation	0.022
	Sig. (2-tailed)	0.862
	N	63
HF (%)	Pearson Correlation	0.395
	Sig. (2-tailed)	0.001
	N	63
LF/HF	Pearson Correlation	0.280
	Sig. (2-tailed)	0.026
	N	63
Omentin-1 (ng/ml)	Pearson Correlation	0.067
	Sig. (2-tailed)	0.602
	N	63
Vaspine	Pearson Correlation	− 0.151
	Sig. (2-tailed)	0.237
	N	63

RHI, reactive hyperemia index; MMSE, Mini Mental State Examination; RMSSD, root mean square of the successive differences in 5 min; SDNN, standard deviation of the NN (R–R) intervals; SD, standard deviation; LF, low frequency; HF, high frequency; LF/HF, low frequency/high frequency

## Discussion

In our current study, we analyzed the relationship between diabetic foot ulcers and sympathovagal balance. Only a few studies have evaluated the sympathovagal balance through HRV analysis in patients with diabetic foot [7]. Furthermore, there are few studies that have evaluated surrogate indices of cardiovascular disease, such as endothelial function indices and serum levels of inflammatory adipokines, in a diabetic population with ulcerative lesions of the lower limbs [1–3].

Some studies by our own group analyzed endothelial dysfunction and adipose inflammatory markers such as omentine and vaspin in subjects with diabetic foot in comparison with those without foot ulcerations [3, 5]. In this actual study we confirmed our previous findings concerning the lower serum levels of omentin-1 lower and the lower median RHI values of subjects with diabetic foot. We also report the association between diabetic foot and omentin-1 and reactive hyperaemia index (RHI) as marker of endothelial dysfunction.

Nevertheless, to the best of our knowledge, no study has yet correlated endothelial dysfunction, assessed noninvasively by means of the RHI, with dysfunction of the autonomic nervous system, assessed by the analysis of HRV, and serum levels of some inflammatory adipokines in a population of patients with diabetes and foot ulcers.

Aso et al. [16] evaluated the sympathovagal balance obtained by means of the spectral analysis of HRV in a population with diabetic foot. In the population examined by Aso, the LF/HF ratio, representing the expression of the sympathovagal balance, was not particularly increased, although both the LF (*sympathetic activation indices*) and the HF (*parasympathetic activation indices*) were decreased.

Our study showed that patients with diabetic foot show a higher degree of activation of the parasympathetic system, expressed by the increase in HF values, compared to diabetic controls and a lower LF/HF ratio compared to diabetic controls. The values of the LF/HF ratio of patients with DFS indicate a sympathovagal balance trending toward a higher degree of parasympathetic activation [17], albeit not statistically significant, consistent with the findings reported by Aso [16].

Takashy et al. [18] noted that patients with diabetes with neuropathy had significant sympathetic dysfunction. Within the pathogenesis of diabetic ulcers, in addition to the already established role of diabetic micro- and macroangiopathy and the impairment of the somatic nervous system, dysfunction of the autonomic nervous system plays an important role. Sympathetic dysfunction involves sweating, dryness of the skin and a reduction in skin thickness, which leads to susceptibility to infections and the formation of a diabetic ulcer from mechanical-compressive damage. Ahmed et al. [19] showed that in patients with diabetic foot, the parasympathetic system is compromised early, which is then followed, over time, by permanent sympathetic dysfunction. Our actual results, depicting greater parasympathetic activation, can be explained by a counterbalance of the vagal action against a reduced sympathetic action.

Our study also highlighted a statistically significant negative correlation between the RHI value and HRV indices and the expression of increased parasympathetic activity (RMSDD and HF%) in subjects with diabetic foot and a statistically significant positive correlation with the LF/HF ratio and the expression of the sympathovagal balance. Endothelial dysfunction, due to excessive oxidative stress and a reduction in NO values, involves the inability of the endothelium to dilate following inducible ischemic stimuli. Endothelial dysfunction and reduced sympathetic activity are closely related in the pathogenesis of diabetic ulcers, as endothelial dysfunction and the consequent microangiopathy of the vasa nervorum involve a downregulation of the small autonomic-sympathetic fibers present in the periphery. This association has also been demonstrated in both healthy and hypertensive subjects by Pinter [20] and Tomiyama [21], respectively.

Few studies have correlated HRV variables and endothelial dysfunction indices. In a previous study by Bedile Irem Tiftikcioglu et al. [22], the correlation between HRV and the RHI was analyzed, but this relationship was not statistically significant. This relationship could be explained by the influence exerted by microangiopathy on autonomic neuropathy. Hyperglycemia can cause changes in the intracellular redox state through the depletion of the cellular NADPH pool. Nonenzymatic glycosylation of proteins and macromolecules secondary to chronic hyperglycemia causes a greater tendency toward oxidative stress and high levels of oxidized lipoproteins (LDL in particular). High levels of fatty acids and hyperglycemia have both been shown to cause an increase in the oxidation level of phospholipids and proteins, resulting in an increased prothrombotic tendency, as well as increased platelet aggregation**.** Numerous prospective studies evaluating endothelial dysfunction in patients with diabetes indicate that it is closely associated with microangiopathy and macroangiopathy [23].

Our finding concerning a positive correlation between the RHI and the LF/HF ratio is therefore a possible original finding, as well as the negative correlation between the RHI and the parasympathetic function indices. It is possible to assume that the persistent reduction in sympathetic activity in patients with diabetic foot can be compensated for at the expense of greater parasympathetic activity to recover the delicate homeostasis of the sympathovagal balance.

Our results did not highlight any particular difference between the percentages of HF and the LF/HF ratio between patients with diabetic foot and healthy patients. A possible explanation is that a discrete percentage of subjects in the control group suffered from chronic ischemic heart disease with consequent use of beta blockers, which caused an increase in parasympathetic activity.

Another interesting result that emerged from our study is the high LF/HF ratio in patients with diabetes compared to DFS and healthy controls. Some studies evaluated HRV in patients with diabetes with or without microvascular complications [22–24]. In most cases, the LF/HF ratio appears to be decreased, especially in patients with diabetic neuropathy [22–24]. Min-Young Chun et al. [25] assessed that worsening of autonomic neuropathy is positively correlated with the consensual reduction in the LF/HF ratio. Eckberg et al. [26] suggested that in the pathogenesis of diabetic neuropathy, there is early and early dysfunction of the parasympathetic system, which is followed by a reduction in the sympathetic system. Me Ahmed et al. [19] proposed a similar pathogenesis within the context of diabetic neuropathy complicated by diabetic foot. Our diabetic cohort examined did not show numerous cases of diabetic neuropathy; thus, it is possible that the LF/HF ratio is increased, indicating an imbalance toward sympathetic activation, precisely due to the early initial parasympathetic dysfunction that characterizes diabetes and that can lead to a rather common microvascular complication such as diabetic neuropathy. Our results show that patients with diabetic foot show significantly lower RHI values and therefore a greater degree of endothelial dysfunction than patients with diabetes without ulcers and healthy controls. Previously, Siasos et al. [27] provided the first evidence regarding the correlation of diabetic ulcerative lesions with endothelial dysfunction. In a recent study by our own group [5], we reported that patients with diabetic foot had a higher degree of endothelial dysfunction, expressed as lower RHI values, compared to patients with diabetes and a control group of healthy patients. A greater degree of arterial stiffness was also found through the analysis of PWV and a greater degree of “mild cognitive impairment”, as assessed by the MMSE scores.

Diabetic foot syndrome (DFS) represents a micro and macrovascular complication of diabetes. Endothelium-dependent vasodilation is markedly impaired in the arteries of patients with hypertension, diabetes, ventricular hypertrophy and other cardiovascular risk factors [28–32]. Therefore, both the micro- and macrovascular complications of diabetes are well represented by the evaluation of the indices of endothelium-dependent vasodilation, such as the RH-PAT index.

Our results showing significantly lower RHI levels in a cohort of patients with diabetic foot compared with a population of patients with diabetes and healthy controls are therefore in line with our previous studies [3, 5] and demonstrate that endothelial dysfunction evaluated with a noninvasive method can be considered a surrogate cardiovascular risk marker.

In a recent cross-sectional observational study [33], conducted on the cohort of patients in the ADELAHYDE has been reported the association of arterial changes (arterial hypertrophy and stiffness, endothelial dysfunction) with cognition indexes Artery thickness and stiffness as well as endothelial function should be measured simultaneously and may represent an additional target for the prevention of memory impairment and WMHs.

In a very recent study by our group [34], worse cognitive performance, a higher prevalence of hyperintense lesions of the white matter on brain MRI, increased PWV values and reduced serum levels of omentin-1 were reported in patients with DFS compared to a diabetic population without ulcers, a healthy population with vascular ulcers and a control population. These patients also had low RHI levels compared to a healthy population with or without mention of a vascular ulcer and comparable RHI values compared to patients with diabetes.

In our study, we have also reported how patients with diabetic foot, compared to diabetic controls without ulcers and healthy controls, have significantly lower serum levels of omentin-1. Recently, in agreement with the results of our group [34], Yamawaki et al. showed how omentin has a vasodilating effect on isolated blood vessels, increasing the production of endothelium-derived NO [35]. The negative association between omentin, circulating IL-6 and C reactive protein was also shown to be related to endothelial dysfunction [36]. The inflammatory state associated with obesity-induced metabolic disorders [37] could be the cause of the dysfunction of endothelial cells in the blood vessels of visceral abdominal tissues, decreasing the expression and production of omentin. Proinflammatory cytokines (TNF-α and IL-6) proportionally indirectly correlate with circulating omentin concentrations [38]. Furthermore, reduced concentrations of omentin in the synovial fluid of patients with rheumatoid arthritis have been described [39]. Inflammation has been widely characterized as an important contributing factor to vascular endothelial dysfunction [40]. Cytokines could induce vasoconstriction by reducing the expression of endothelial NO synthase, reducing the bioavailability of NO and inducing the synthesis of endothelin-1 [41, 42]. Reduced expression of omentin in omental fat endothelial cells in patients with visceral obesity may reflect the dysfunction of these cells caused by an obesity-associated proinflammatory state and oxidative stress.

Thus, the concentration of circulating omentin in patients with IGT could be a useful biomarker of endothelial function.

Vaspine is an inflammatory adipokine produced by visceral and subcutaneous human adipose tissue [43] and appears to act on the adipoinsulin axis and to be associated with insulin resistance, especially in patients with type 2 diabetes mellitus [44]. Although vaspine has shown its ability to improve glucose tolerance and insulin sensitivity in mice, in humans, it seems to be positively associated with obesity-related diseases [45]. Some evidence shows that vaspine levels can change according to the progression of diabetic disease; in particular, they increase at the onset of the disease and decrease later on [46].

Our results showing non significant differences between the three groups of vaspine serum levels and the significantly lower serum levels of omentin-1 in patients with diabetic foot compared to diabetic and healthy controls can be explained by the different pathogenetic roles of these two adipokines in the context of microvascular foot damage. A recent study [47] reported a significant correlation between serum vaspine levels and leptin concentrations, indicating that serum vaspine levels are a reflection of the amount of human adipose tissue and that vaspine may play a compensatory role in insulin resistance, the main pathogenesis of diseases related to obesity. Our diabetic foot patients, compared to diabetic and healthy controls, had comparable BMI values and more or less equal body weights, which may be sufficient to explain why serum vaspine concentrations did not vary among the three groups.

## Limitations

The cross-sectional analysis is a limitation because it does not allow conclusions on cause–effect relationships, and the relatively small sample size may also limit conclusions.

## Conclusions

Thus, we can conclude that evaluation of heart rate variability (HRV) may be advisable in patients with diabetic foot because the presence of foot complications in these subjects seems to make these patients more prone, in comparison with subjects with diabetes without foot complications other vascular complications [48]. Our findings may corroborate the issue that a parasympathetic dysfunction may have a possible additive role in the pathogenesis of other vascular complications in subjects with DFS.

## Data Availability

All data and material are available on figshare (https://figshare.com/s/e5faff6f3810505292c9).

## Abbreviations

HRV: Heart rate variability
ECG Holter: Electrocardiogram Holter
RMSSD: Square root of the mean of successive differences of NN (), standard deviation or square root of the variance (SD)
SDANN/5 min: Standard deviation of the means of the NN intervals calculated over endo-PAT, and serum levels of vaspine and omentin-1 were assessed by blood sample collection
NO: Nitric oxide
DFS: Diabetic foot syndrome
WHO: World Health Organization
NSS: Neuropathy Symptom Score
ADA: American Diabetes Association
ESC: European Society of Cardiology
HDL: High density lipoprotein
BMI: Body mass index
MMSE: Mini-Mental State Examination
RH-PAT: Reactive hyperaeamia peripheral artery tonometry
RHI: Reactive hyperactivity index
HR: Heart rate
SVT: Supraventricular tachycardia () (HR > 150 bpm)
TV: Ventricular tachycardia
SVBE: Supraventricular ectopic beats
EVB: Ventricular ectopic beats
RMSSD: Square root of the mean of successive differences of NN
SD: Standard deviation or square root of the variance
SDANN/5 min: Standard deviation of the means of the NN intervals calculated over a five-minute period
HF%: High frequency
LF%: Low frequency
LF/HF ratio: Lower frequency/high frequency
SDs: Standard deviations
ANOVA: Univariate analysis of variance
WMH: White matter hyperintensity
PWV: Pulse wave velocity
TNF-α: Tumor necrosis factor alfa
IL-6: Interleukin 6
